# The Beat to Read: A Cross-Lingual Link between Rhythmic Regularity Perception and Reading Skill

**DOI:** 10.3389/fnhum.2016.00425

**Published:** 2016-08-31

**Authors:** Annike Bekius, Thomas E. Cope, Manon Grube

**Affiliations:** ^1^Machine Learning Group, Technische Universität BerlinBerlin, Germany; ^2^Department of Human Movement Sciences, Institute Brain and Behaviour, Vrije Universiteit AmsterdamAmsterdam, Netherlands; ^3^Auditory Group, Newcastle UniversityNewcastle-upon-Tyne, UK; ^4^Department of Clinical Neuroscience, University of CambridgeCambridge, UK

**Keywords:** regularity, rhythm, auditory, timing, beat, language, cross-lingual, reading

## Abstract

This work assesses one specific aspect of the relationship between auditory rhythm cognition and language skill: regularity perception. In a group of 26 adult participants, native speakers of 11 different native languages, we demonstrate a strong and significant correlation between the ability to detect a “roughly” regular beat and rapid automatized naming (RAN) as a measure of language skill (Spearman's rho, −0.47, *p* < 0.01). There was no such robust relationship for the “mirror image” task of irregularity detection, i.e., the ability to detect ongoing small deviations from a regular beat. The correlation between RAN and regularity detection remained significant after partialling out performance on the irregularity detection task (rho, −0.41, *p*, 0.022), non-verbal IQ (rho, −0.37, *p* < 0.05), or musical expertise (rho, −0.31, *p* < 0.05). Whilst being consistent with the “shared resources model” in terms of rhythm as a common basis of language and music, evolutionarily as well as in individual development, the results also document how two related rhythm processing abilities relate differently to language skill. Specifically, the results support a universal relationship between rhythmic regularity detection and reading skill that is robust to accounting for differences in fluid intelligence and musical expertise, and transcends language-specific differences in speech rhythm.

## Introduction

The existence of a general relationship between language skill and auditory processing is widely accepted (Lukens, 1896). We typically acquire a language by first listening to it, and then by speaking it, before developing reading and writing skills (Harris, 1947). The perception of rhythm is emerging as being particularly relevant to both the normal acquisition of language skill and disorders of language (e.g., Huss et al., 2011; Grube et al., 2012, 2013, 2014; Przybylski et al., 2013; Gordon et al., 2015; Wieland et al., 2015).

The behavioral link between speech and language skills on the one hand, and aspects of temporal processing on the other, has been attracting research interest in recent decades. Early studies used single sounds or sound pairs, demonstrating links with reading skill, or language impairments for temporal processing ability at the segmental time scale of individual phonemes (Tallal, 1980; Wright et al., 1997; Goswami et al., 2002; Walker et al., 2006; Moore et al., 2010). However, language typically comes in sentences, i.e., streams of syllables consisting of several phonemes over a period of seconds, typically with a characteristic rhythm and stress pattern. The theory that children first process the whole sentence or phrase before breaking it down into single phonemes (Metsala and Walley, 1998) underpins the need to explore the role of sequence processing at supra-segmental time scales. Speech rhythm work in infants and adults has documented the relevance of rhythmic cues and durational patterns, in particular word and phrase boundaries (Smith et al., 1989; Jusczyk et al., 1992) that manifest in the “quasi-rhythmic” (Giraud and Poeppel, 2012) temporal structure of speech (Rosen, 1992).

Recent studies on auditory processing and language or literacy skills have provided evidence for a long neglected role for rhythm and timing *per-se*, rather than simply for the processing of acoustic features, such as pitch, over time. Huss et al. (2011) reported group-level deficits for the detection of changes in musical rhythms of varying meter in dyslexic compared to control children, and a significant correlation between rhythm and phonological and literacy measures across groups. Using a number of rhythm and timing (as well as pitch and timbre) measures, our work in a large cohort of 11-year olds (Grube et al., 2012) demonstrated correlations that were most robust and least affected by general intellectual skill between language and literacy skills and the rhythm domain. The strongest and most consistent correlation (with a Spearman's rho of about 0.3) was found for the processing of short, isochronous 5-tone sequences (1.5–2.5 s), corresponding to short sentence or phrase levels in speech. Less consistent correlations with language and literacy skill were found for the more musically oriented detection of perturbations in a longer, strongly metrical rhythmic sequence (composed of 7 tones, with an average duration of 3.2 s), created by equidistant spacing of accented tones in time (Grube and Griffiths, 2009). In another study we looked specifically at the processing of different types of longer rhythmic sequences and reading skill in young, English-native speaking adults (Grube et al., 2013). We found a strong and consistent correlation (with a Spearman's rho of about 0.5) for the detection of a “roughly regular” beat, created by adding a parametrically varied amount of jitter to 11-tone sequences with an average length of 4.4 s, corresponding to sentence levels in speech. Notably, this correlation was not seen in the 11-year olds, suggesting a relevance for this ability in later, higher-order language development. The second strongest correlations in both studies were found for the detection of perturbations in a strongly metrical rhythm. Whilst the metrical rhythms represented a highly simplified version of the hierarchical time structure of Western music (London, 2004), the roughly regular rhythms might similarly mimic the quasi-rhythmic structure of speech (Giraud and Poeppel, 2012). The regularity detection task might therefore capture an ability relevant to speech. This task measures the point at which the participant cannot reliably tell which of two simple tone sequences is closer to being regular, i.e., less random. In the beginning, one sequence is perfectly regular (isochronous), the other one highly irregular (by adding a jitter of 30%). Over the course of the task, the initially regular sequence becomes less and less regular, until both sound equally irregular to the listener. The previously demonstrated correlation with reading skill in English-speaking young adults (Grube et al., 2013) suggests a role specifically for this ability to “pull out” such a just noticeable regularity at the sequence (i.e., sentence) level, beyond the sound of single phonemes, and relevant to higher-order language skills. Why would this be? We argue that the subjectively perceptible, somewhat regular rhythm of speech is similar in temporal structure to the “rather irregular” rhythms with an intermediate to high jitter used here. Specifically, sequences with a jitter of up to 15% are closest to the variability of syllable duration and inter-stress intervals in speech, and the presence of regularities in this range aid speech perception (Tsyplikhin, 2007). We demonstrate sensitivity to regularities within an irregular sequence in the same range, and our inter-onset-intervals, with an average of 400 ms, would correspond to the inter-stress intervals, equivalent to the temporal separation of every second or third syllable (Scott, 1982; Rosen, 1992; Grabe and Low, 2002; Tilsen and Arvaniti, 2013).

The aim of this work is to test for a dissociated pattern in correlation with adult reading skill for this ability to extract such a roughly regular beat, compared to the related “mirror image” ability to detect small deviations from a perfectly regular beat.

Both tasks start out with one sequence being perfectly regular, and one being highly irregular. In both tasks, the difference between the two becomes progressively smaller, in an adaptive manner according to individual performance. A listener's perceptual threshold is defined here as the point at which they are able to correctly distinguish the sequences 70.9% of the time (Levitt, 1971). Nonetheless, the tasks fundamentally differ; the irregularity detection task tests for the smallest perceivable distributed deviation from perfect isochrony, while the regularity detection task tests for the largest degree of irregularity at which the listener is able to perceive any regularity at all. Phenomenologically, in the irregularity detection task the listener attempts to distinguish increasingly regular sequences, whilst in the regularity detection task the listener chooses between increasingly irregular sequences.

With respect to the underlying mechanisms of timing, we hypothesize that performance in the two tasks relies on differential contributions of two or more complementary mechanisms of “absolute,” (i.e., duration-based) and “relative,” (i.e., beat-based) timing (Grube et al., 2010a,b; Breska and Ivry, 2016: “discrete vs. continuous”; Teki et al., 2011). For the irregularity detection task, toward the end of which the listener is presented with two seemingly isochronous sequences, we expect performance to rely largely on beat-based timing mechanisms, supported by the striato-thalamo-cortical subsystem (Teki et al., 2011). For regularity detection in contrast, in which the listener will be facing two highly irregular sequences, we would expect performance to depend more on duration-based timing mechanisms, supported by the olivo-cerebellar sub-system (Teki et al., 2011). The two tasks also differ in whether a comparator beat is provided to the listener or must be generated internally, reinforcing the distinction between cerebellar and basal ganglia dependence (Grahn, 2009).

The available data therefore necessarily support a partial dissociation of the two subsystems responsible for the processing of regular and irregular sequences. Functionally, we argue that they contribute differentially to absolute vs. relative timing in the subsecond range (Chen et al., 2008; Grahn, 2009; Teki et al., 2011), relevant to language and music. In terms of underlying mechanisms, recent neurophysiological work has implicated neural oscillations in the theta, alpha, and beta ranges as playing a key role in entrainment with the temporal patterns of regular or metrical beats (Iversen et al., 2009; Fujioka et al., 2012), as well as those of pseudo-regular speech envelopes (Ghitza, 2011; Wöstmann et al., 2016). Consistent with domain-general timing functions, neuroimaging studies on shared brain bases for music and speech have demonstrated a common network involving middle and superior temporal gyri and inferior and middle frontal gyri (Schön and Tillmann, 2015). The similarities in brain bases between music and speech, especially in the temporal domain, further motivate our search for universal behavioral correlations.

In terms of our everyday lives, we hypothesize that the ability to pull out an ever-so-roughly regular beat from highly irregular sequences plays a critical role in speech perception and production. In contrast, we would expect sensitivity to small deviations from an isochronous beat (irregularity), to be less relevant to the successful processing of “quasi-rhythmic” speech.

Furthermore, we postulate that a behavioral relationship between regularity detection and reading skill would reflect a universal biological relationship. We argue that the ability to detect a roughly regular beat is a sensitive measure for a “temporal scaffolding mechanism” that supports the perception and production of any language, despite possible differences in speech rhythm. We therefore test here for a correlation between the two rhythm cognition measures of regularity and irregularity detection and rapid reading skill in a mixed cohort of different native language speakers. We assessed reading skill with the rapid automatized naming task (RAN) from the York Adult Assessment Battery of phonological and literacy skill. This is a standardized test that is a validated predictor of literacy skills (Warmington et al., 2013). We specifically chose RAN as a measure of fast reading that can be easily and comparably applied in different mother tongues (Georgiou et al., 2008).

In continuation of the findings leading up to this study (Grube et al., 2013), this work assesses the following novel aspects of the association between auditory rhythm perception and reading skill: (i) the constancy of the correlation with regularity detection across languages and a wider age range; (ii) the dissociation in correlation for an irregularity detection task using similar sequences.

## Materials and methods

The order of behavioral testing was the same as the order of tasks in this methods section. Total session duration was approximately 45 min. Participants were instructed in English, the one common language of proficiency between all participants and experimenters, but performed the RAN test in their mother tongue.

### Participants

The study was conducted in 26 adults (age range 20 to 40, mean age 28 ± 4.6 years; 12 male), who were native speakers of 11 different mother tongues (Danish, 1; Dutch, 3; German, 11; English, 2; French, 1; Greek, 1; Italian, 1; Romanian, 1; Slovenian, 2; Spanish, 1; Turkish, 2). They were in part professionals and in part students from different disciplines; duration of education ranged from 13 to 27 years (mean, 19.1 years ±3.7). Musical expertise ranged from none to (semi/ex) professional, summarized in a score on a scale 1–5, based on the amount of musical training: 1, no musical experience; 2, up to three years of practice; 3, more-than-three to eight years of practice; 4, more than eight years of practice; 5, professional musicianship. Table 1 contains individual demographics and descriptive group statistics. The study was in accord with the guidelines of, and approved by, the Ethics Committee of the Department of Psychology at TU Berlin. All subjects gave written informed consent in accordance with the Declaration of Helsinki.

**Table 1:** **Individual scores and descriptive group statistics for all measures: fluid intelligence, musical expertise, the two auditory timing tasks, and rapid reading**.

**Subject number**	**Languages**			**Musical expertise**				**WASI matrices (raw score, 0–35)**	**Regularity detection threshold (%)**	**Irregularity detection threshold (%)**	**RAN time (sec)**
	**Mother tongue**	**Proficient/advanced**	**Beginner**	**Years of practice**	**Starting age**	**Instrument**	**Score (1–5)**				
1	Dan	Eng	Ger	0	–	–	1	29	17.5 ± 2.2	3.8 ± 1.0	13.78
15	Dut	Eng, Ger		9	8	Flute, piano	4	31	19.3 ± 3.4	3.8 ± 1.2	13.25
18	Dut	Eng, Ger	Tur	15	8	Piano, vocal	4	29	19.7 ± 4.1	3.3 ± 1.4	10.24
19	Dut	Eng, Ger		7	9	Recorder, flute	3	31	19.9 ± 4.6	6.0 ± 1.3	12.39
4	Eng	Spa, Ger		3	11	Piano	2	31	15.2 ± 5.4	3.7 ± 1.4	11.02
13	Eng	Ger	Jap	8	6	Piano, vocal	4	29	24.0 ± 3.4	2.3 ± 1.4	14.36
17	Fre	Ger, Eng		4	8	Piano	3	26	16.7 ± 4.7	4.7 ± 2.1	15.52
3	Ger	Eng		20	5	Cello	5	28	21.6 ± 4.3	2.3 ± 0.7	13.13
5	Ger	Spa, Eng	Fre	12	6	Piano	4	32	17.1 ± 5.1	2.3 ± 1.0	13.91
7	Ger	Eng	Fre, Swe	6	12	Cello, guitar	3	26	10.5 ± 2.2	4.5 ± 0.5	13.63
9	Ger	Eng		10	8	Violin, piano, drums, guitar	4	30	25.0 ± 3.5	3.3 ± 1.2	12.31
11	Ger	Eng		9	10	Piano	4	32	20.0 ± 2.2	2.8 ± 1.0	10.29
12	Ger	Eng		13	11	Keyboard, guitar, vocal, choir	4	26	11.1 ± 4.3	6.2 ± 1.2	15.52
14	Ger	Eng, Fre		11	3	Recorder, clarinet	4	33	21.3 ± 3.8	8.5 ± 1.0	11.84
21	Ger	Eng	Fre, Spa	–	–	–	1	30	14.7 ± 4.9	2.7 ± 1.2	14.40
23	Ger	Eng		0.5	13	Piano	2	29	18.1 ± 4.3	7.0 ± 1.5	13.17
24	Ger	Eng		9	8	Piano, drums	4	32	23.2 ± 3.8	4.7 ± 2.0	14.27
26	Ger	Eng		10	8	Saxophone	4	28	24.2 ± 3.8	2.0 ± 2.3	17.57
20	Gre	Ger, Eng, Fre		10	8	Guitar	4	31	21.3 ± 4.7	2.7 ± 0.8	13.94
6	Ita	Eng		2	10	Not known	2	28	9.9 ± 2.9	14.0 ± 4.8	14.57
2	Rom	Eng, Fre		0	–	–	1	27	16.5 ± 0.7	4.2 ± 1.2	14.81
22	Slo	Eng, Cro		18	13	Guitar	5	24	16.8 ± 2.0	4.3 ± 1.4	16.34
25	Slo, Ser, Cro	Eng, Ger, Spa		18	4	Violin, vocal, choir	5	32	14.4 ± 6.6	4.2 ± 1.2	13.53
16	Spa	Eng, Ger, Cat		0	–	–	1	28	15.3 ± 3.4	7.7 ± 1.9	19.11
8	Tur	Eng, Ger	Dut	4	12	Vocal, choir	3	31	14.8 ± 3.0	4.0 ± 1.3	16.36
10	Tur	Ger, Eng		13	6	Piano, guitar, drums	5	31	27.1 ± 2.9	2.0 ± 1.1	9.92
Mean ± SD				8.1 ± 6	8.5 ± 2.8		3.3 ± 1.3	29.3 ± 2.1	18.3 ± 4.5	4.5 ± 2.6	13.9 ± 2.3

*NB, for the irregularity detection task, lower threshold values indicate better performance, i.e., closer to the isochronous reference jitter of 0%, while for the regularity detection task, higher threshold values indicate better performance, i.e., closer to the irregular reference jitter of 30%. WASI, Wechsler Abbreviated Scale of Intelligence; RAN, Rapid Automatized Naming; SD, Standard Deviation. Languages: Cat, Catalonian; Cro, Croatian; Dan, Danish; Dut, Dutch; Eng, English; Fre, French; Ger, German; Ita, Italian; Jap, Japanese; Rom, Romanian; Slo, Slovenian; Ser, Serbian; Spa, Spanish; Swe, Swedish; Tur, Turkish*.

### Test of fluid intelligence

Fluid intelligence was measured using the progressive matrices from the Wechsler Abbreviated Scale of Intelligence (WASI), scored as the number of items correct. We used raw scores rather than standard scores, as all participants would be in the same age group. (Any non-linearity in the transformation into standard scores is thus not included but would in any event have no effect on the non-parametric correlation analyses employed here.)

### Reading task

Reading skill was measured by the digit version of the standardized rapid automatized naming test (RAN) from the Revised York Adult Assessment Battery (Warmington et al., 2013). The task is to read a list of 50 digits as fast as possible, after a short practice of 7 items. The outcome measure is the time needed (in s) to read the full list of 50. Each participant performed the task in their mother tongue.

### Auditory rhythmic timing tasks

#### Setup

Testing was performed in a quiet room. Tasks were implemented in Matlab, 2012b. Stimuli were created at 44.1-kHz sampling rate (16-bit resolution), delivered via an external soundcard (Edirol UA-4FX) and closed headphones (Sennheiser HD 380 pro) at approximately 80 dB rms sound pressure level.

#### Stimuli

Sequences were composed of nine to eleven 300-Hz pure tones (each of 100 ms duration including 20 ms raised cosine ramps), and using one out of three possible underlying tempi to avoid habituation and learning effects. The three possible tempi had mean inter-onset-intervals of 340, 400, or 460 ms; deviations from this pulse rate depended on the degree of irregularity applied to each sequence. The length and tempo of the sequences were carefully piloted and chosen to “sound right,” and have been previously validated in developmental (Grube et al., 2012, 2014), neurodegenerative (Grube et al., 2010a; Cope et al., 2014a,b), and healthy adult (Grube et al., 2010b, 2013) cohorts. The sequences were sufficiently long to allow the perceptual judgment about the presence of a “roughly” regular beat, whilst being sufficiently short that listeners did not get bored. Tempi were chosen to be within the optimal range for the perception of a beat (Fraisse, 1984; Drake et al., 2000; Grondin, 2001; London, 2004).

#### Tasks

Auditory rhythmic timing was assessed by two tasks, one measuring the ability to detect small deviations from a perfectly regular sequence, and the other to detect a roughly regular beat within a highly irregular sequence. Both tasks were based on an adaptive two-alternative forced-choice paradigm. One trial consisted of two sequences (reference and target) presented in pseudo-randomized order. Subjects indicated the perceived target position by pressing 1 or 2 on a standard keyboard. Target-to-reference differences were supra-threshold initially, and were adaptively adjusted according to individual performance following a two-down-one-up algorithm, with a convergence level at the 70.9% correct point of the psychometric function (Levitt, 1971). The algorithm used a larger step size up to the fourth reversal and after that a smaller one.

The parametrically varied feature of interest is the degree of irregularity (“jitter”), introduced to the sequences by shifting each tone forward or backward. The jitter values used range from 0% (perfectly regular; isochronous) to 30% (highly irregular), and are realized by pseudo-randomly shortening and lengthening each individual inter-onset-interval by the desired jitter value ±50%. For a jitter value of 30% for instance, inter-onset-intervals were changed by 15–45%, in a way that the average change across the sequence was 30%. For details on additional constraints to avoid accidental interval repetitions etc. see Cope et al. (2014a); example sequences are available to listen to in Supplementary materials.

**Figure 1:**
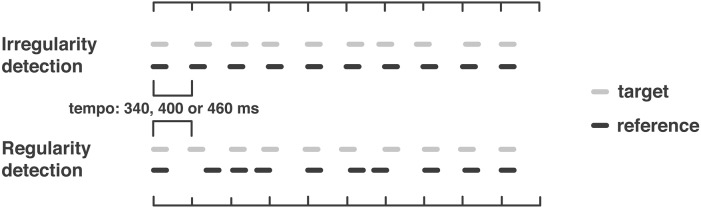
**Schematic depiction of auditory timing tasks: (top) irregularity detection; (bottom) regularity detection**. In both tasks, the target becomes more and more like the reference, only that this means in one case to listen to two highly regular sequences toward the end of the task (irregularity detection), and to two highly irregular sequences (regularity detection). Depicted are one exemplar reference and one target per task.

In the *irregularity detection task* (Figure 1, top; adapted from Cope et al., 2014a,b) subjects were required to indicate which of the two sequences presented per trial was more “irregular.” The reference sequence is perfectly regular (0% jitter) throughout. The target has a clearly noticeable jitter of 20% initially, which is then adaptively adjusted in steps of 3 and 1% according to performance. Over the course of the task, the target approaches the reference jitter (0%) until the point at which the subject cannot detect which of the two sequences is not perfectly regular.

In the *regularity detection task* (Figure 1, bottom; adapted from Grube et al., 2010a,b, 2012, 2013, 2014; Cope et al., 2014a,b) subjects were required to indicate which of two sequences was more “regular.” In this task, the reference is always highly irregular (30% jitter). The target is initially perfectly regular (0% jitter), and is adaptively adjusted in steps of 4 and 2.5%. Over the course of the task, the target approaches the reference jitter (30%) to the point at which the subject cannot detect which sequence contains more regularity.

Total number of trials per task was 48. Thresholds were calculated as the mean of the last 6 reversals (of the small step size). Inter-stimulus intervals (from the end of the first to start of the second sequence within each trial) and inter-trial intervals (from response to the start of the first sequence of the next trial) were 1500 ms each. The tasks took about 15 min each.

Task order was fixed in the way that made the most sense in terms of leading the subject through the session: starting with matrices (progressing from easy to harder ones, and also, although being the control measure, the longest task); followed by RAN (fast and fun, but best not performed “out of the cold”); and then the two timing tasks: firstly the irregularity detection (easily grasped) and secondly the regularity detection task (a little more unusual and best understood second; see online example stimuli).

### Statistical data analysis

Due to significant deviations from normal distribution (Table 2), revealed by the Lilliefors version of the Kolmogorov-Smirnoff Test, correlation analysis used Spearman's rho. The one-tailed version was used based on the a-priori hypothesis of a positive correlation between performance on reading and rhythm tasks. In a second step, in order to control for potentially confounding effects of musical expertise and non-verbal IQ, scores for musical expertise and WASI matrices were partialled out (of the correlations between RAN and rhythm measures). As the two rhythm tasks shared some underlying variance, the dissociation of relationship to language skill was finally confirmed by examining each task partialled out of its counterpart.

**Table 2:** **Correlation strength and significance between auditory timing abilities and rapid automatized naming skill, and the effects of musical expertise and fluid intelligence**.

	**RAN**	**RAN with mirror task partialed out**	**Musicality**	**RAN with musicality partialed out**	**Matrices**	**RAN with matrices partialed out**	**RAN with musicality and matrices partialed out**
Irregularity detection	0.26, 0.101	(0.03, 0.447)	**−0.39**, 0.026	(0.16, 0.225)	(−0.19, 0.181)	(0.19, 0.187)	(0.12, 0.292)
Regularity detection	**−0.47**, 0.008	**−0.41**, 0.022	**0.49**, 0.006	**−0.38**, 0.029	0.32, 0.057	**−0.37**, 0.036	−0.31, 0.071

*Listed are Spearman's rho correlation coefficients and p-values for correlations of the two timing tasks with: the rapid reading measure (RAN, time needed in s), followed by the same, but with accounting for shared variance between the two timing tasks; the covariate of musical expertise (score from 1 to 5), followed by the partial one with RAN, after controlling for musical expertise; the covariate of fluid intelligence (WASI matrices scores), followed by the partial correlation with RAN, after controlling for fluid intelligence; and RAN, after partialing out musical expertise and matrices. NB: Some correlations have a negative and some a positive sign, depending on the combination of measures. Irregularity and RAN measures are “the lower the better”; regularity, musical expertise, and matrices measures are “the higher the better.” Whilst not all correlations are significant, all of them, even those that only show a trend, are consistent with the direction of performance being positively correlated. Note how strong and significant the correlation with RAN time needed is for regularity compared to irregularity detection thresholds. This difference becomes even clearer when partialing out (of the correlation with RAN) the shared variance for any of the other measures. Significance level was p < 0.05. In bold, significant. In brackets, Spearman's rho < 0.22 (explaining < 5% variance)*.

## Results

### Correlations between auditory timing tasks, musical expertise, and fluid intelligence

As expected, the two timing tasks of regularity and irregularity detection themselves were strongly and significantly correlated with each other (Spearman's rho, −0.5, *p*, 0.004). Irregularity detection thresholds ranged from 2 to 14% (median, 3.9%) jitter, regularity detection thresholds from 9.9 to 27.1% (median, 17.8%) jitter. Note that for irregularity detection, lower threshold values indicate better performance, whilst for regularity detection higher thresholds are better, hence the negative Spearman's rho correlation coefficient between the two tasks.

Performance on both timing tasks was also positively correlated with musical expertise, and this effect was somewhat stronger for regularity detection (rho, 0.49, *p*, 0.006) than irregularity detection (rho, −0.39, *p*, 0.026). After partialling out the effect of musical expertise, the correlation between the two timing tasks was somewhat reduced in strength but remained significant (rho, −0.39, *p*, 0.026).

Neither of the timing tasks was significantly correlated with fluid intelligence, although the relationship trended toward significance for regularity detection (rho, 0.32, *p*, 0.057) whilst being very weak for irregularity detection (rho, −0.19; *p*, 0.18). The correlation between the two timing tasks was virtually unaffected by partialling out the effect of fluid intelligence (rho, −0.48, *p*, 0.008).

### Correlation with reading skill

**Figure 2:**
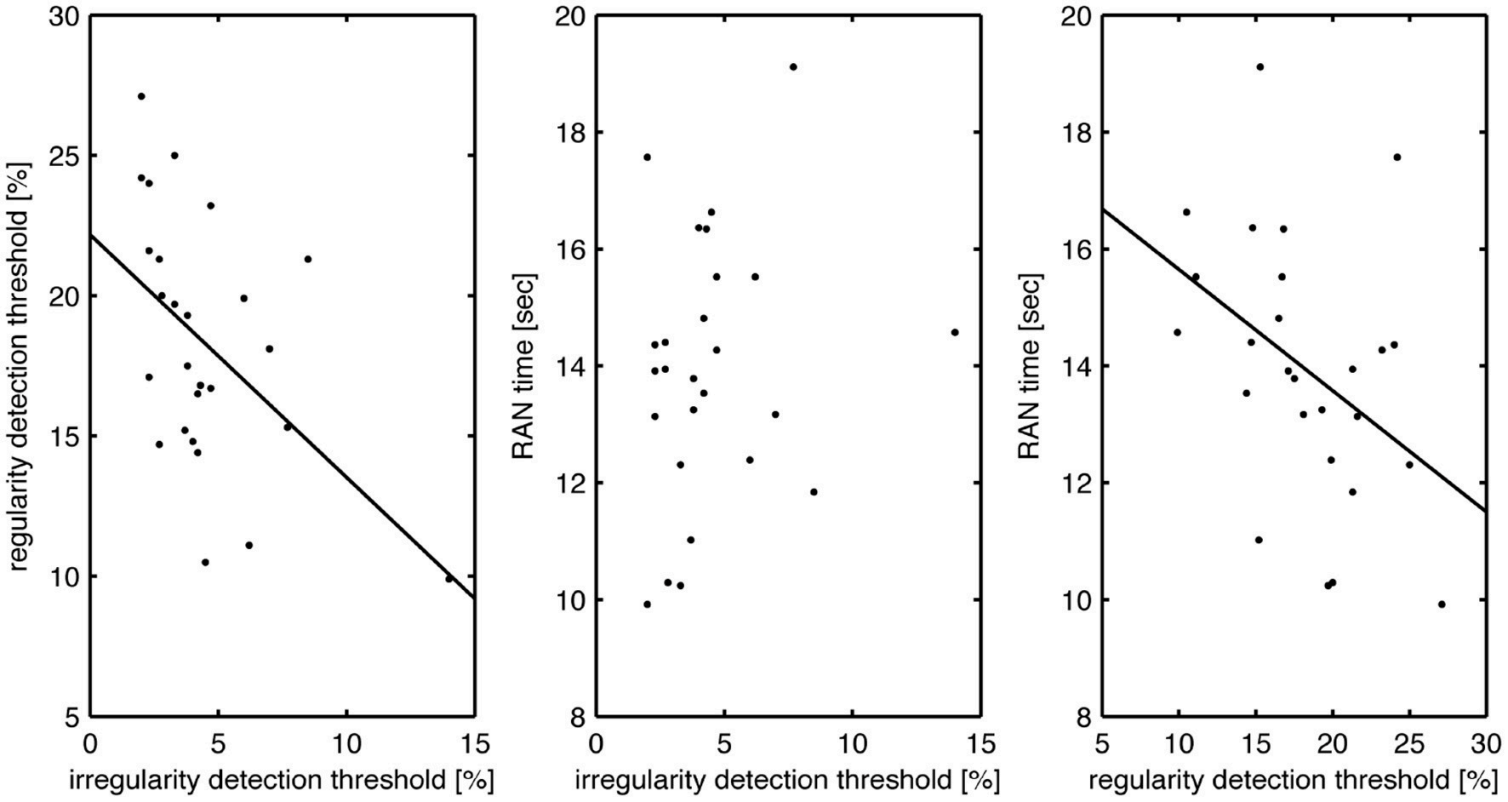
**Correlations between auditory timing abilities and reading skill**. Left, regularity thresholds as a function of irregularity thresholds (significant: rho, −0.5, *p* < 0.001); middle, RAN scores as a function of irregularity detection thresholds (not significant: rho, 0.26, *p* > 0.1); right, RAN scores as a function of regularity detection thresholds (significant: rho, −0.47, *p* < 0.01).

In support of the central hypothesis, there was a strong, statistically significant correlation between rapid automatized naming (RAN) scores and regularity detection thresholds (rho, −0.47, *p*, 0.008; Table 2, Figure 2). Note that the correlation coefficient is negative as better performance is indicated by larger regularity detection thresholds but lower RAN times. This correlation remains significant after partialling out the effect of musical expertise (rho, −0.38, *p*, 0.029) or fluid intelligence (rho, −0.37, *p*, 0.036), and borderline significant after partialling out both (rho, −0.31, *p*, 0.072). In other words, the correlation between regularity detection and RAN explained 22% of the variance before, and 10% after, partialling out the effects of both musical expertise and fluid intelligence.

Strikingly, there was no such strong or significant correlation between irregularity detection thresholds and rapid automatized naming (RAN) scores. There was a weak trend for positive correlation of performance (rho, 0.26, *p*, 0.102), which became weaker still when partialling out musical expertise (rho, 0.16, *p*, 0.225), matrices scores (rho, 0.19, *p*, 0.187) and both together (rho, 0.12, *p*, 0.292).

Given the strong correlation between the two timing tasks, we also tested their partial correlations with RAN time needed, whilst accounting for their shared variance. This yielded a Spearman's rho correlation coefficient of −0.41 (*p* = 0.022) for regularity detection, compared to a rho of 0.03 (*p* = 0.447) for irregularity detection thresholds. This dissociation confirms that individual RAN speed is significantly more strongly related to perceptual threshold for regularity detection than irregularity detection.

Finally, we separately tested the correlations for RAN times against fluid intelligence and musical expertise. Consistent with the effects of partialling these out of the *RAN* correlations with regularity and irregularity detection, there was a strong correlation with RAN for the matrix reasoning scores (rho, −0.59, *p*, 0.0007) and a moderate correlation of borderline statistical significance for musical expertise (rho, −0.31, *p*, 0.06).

## Discussion

The present work comprises a test of two, complementary, “mirror image” aspects of auditory rhythm cognition, and their relationship with reading skill. Both rhythm cognition measures are based on simple tone sequences of varying degrees of regularity. One measures the ability to detect small ongoing deviations from an isochronous beat in highly regular sequences (irregularity detection); the other measures the “opposite” ability in terms of the spectrum of regularity processing; that is, the ability to extract a just noticeable, “roughly” regular beat from highly irregular sequences (regularity detection).

### The “roughly regular” beat to read

The central hypothesis in this work is that the ability to track a “roughly” regular beat is a key ability for the development of speech and language skills, which is universal across different languages. Consistent with this hypothesis, the results demonstrate a significant, robust and specific correlation between the detection of a roughly regular beat and rapid number reading (RAN). They further demonstrate a lack of such a relationship for the “mirror opposite” perceptual timing ability to detect ongoing deviations from a perfectly regular beat.

The regularity detection task objectively measures the listener's threshold in terms of the just noticeable degree of regularity, rather than the subjective judgment inherent in explicitly asking the listener whether or not they thought a sequence had an underlying regular beat. It uses an adaptive, criterion-free, 2-alternative, forced-choice paradigm in which the reference sequence always has a jitter of 30%, which renders the beat imperceptible (Madison and Merker, 2002). Toward the end of the task both sequences within a trial sound highly irregular to the listener and the objective is to decide which one is just that little bit closer to being regular.

The irregularity task measures the opposite ability; namely the ability to perceive small deviations from an isochronous beat, whereby one sequence is always perfectly regular. Toward the end of this task, both sequences sound very regular, and the subject is asked to decide which one contains ever-so-small deviations from perfect isochrony. Both abilities are related to the processing of regularity, and the two measures are correlated (rho, −0.5, *p* < 0.001), yet, importantly, they show a dissociated pattern of correlation with reading skill.

Our previous work has assessed the relationship between rhythm processing and sentence-level reading tasks (Grube et al., 2013). The present study extends this to RAN, a measure of reading in the wider sense (Di Filippo et al., 2005) and finds an even stronger correlation, specifically for the detection of a just noticeable degree of regularity. In other words, the ability to detect a roughly regular beat, similar to the quasi-rhythmic temporal structure of speech, correlates with the ability to rapidly read a page-long list of digits. When broken down into its cognitive constituents, RAN performance relies to a great extent on the strength of connection between orthographic and phonological representations, articulatory fluency, working memory, and the capacity to make rapid eye movements (saccades; Norton and Wolf, 2012). As our participants were all relatively young adults, and none had a disease of the nervous system, we would expect their saccadic latencies and velocities to be similar (Carpenter and Williams, 1995; Antoniades et al., 2007). We are interested in RAN as one measure of fast reading, and the correlation with rhythmic processing. How far this relationship can be broken down to the factors contributing to RAN speed cannot be known. We did not assess those factors, but would predict that further work might demonstrate a particular correlation between rhythm processing and articulatory agility, fluency, and working memory. Whether individuals who are better at detecting rhythmic regularities and able to read out loud faster also tend to read out aloud in a more rhythmically regular fashion will be subject of future work.

The data are consistent with a shared cognitive “sequencing” mechanism (Tillmann, 2012) for structuring events in time, both for rhythmic auditory input and motor speech output. Importantly, this relationship is present across languages with different rhythmic structures, supporting this being a universal mechanism for language acquisition.

Notably, and despite a significant correlation of fluid intelligence with reading skill and a marginally significant one with regularity detection, the relationship between reading skill and regularity detection could only be explained to a small degree by non-verbal intelligence as measured by progressive matrices. This is consistent with our previous findings, in which the correlations between rhythm processing and language skills were relatively independent of non-verbal intellectual skill in early adolescence and early adulthood (Grube et al., 2012, 2013). That is, these correlations were less affected by partialling out the effects on non-verbal IQ than those for pitch processing or processing speed (e.g., Deary, 1994; Stewart et al., 2015).

### Generic regularity and its relationship to musical and speech rhythm

The interval durations were chosen to be within the optimal range for the detection of a regular beat (Fraisse, 1984; Drake et al., 2000; Grondin, 2001; London, 2004). At the same time, these durations match the time scale between supra-segmental markers and stressed syllables or “beat intervals” in spoken speech (Scott, 1982; Rosen, 1992; Grabe and Low, 2002; Tilsen and Arvaniti, 2013). Each sequence had a unique jitter pattern, mimicking natural speech, in which no two sentences are identical. At the same time, the chosen tempi correspond well to those used in Western music. Notably, and in contrast to speech, musical rhythm (the succession of events in time) and meter (the underlying beat) are typically precisely defined and predictable, building on an isochronous beat and featuring an hierarchically organized metrical structure with nested levels of periodicity (London, 2004). We would argue that whilst the irregularity detection task, which is based on detecting small deviations from isochronous sequences, tested an aspect of perceptual timing more relevant to musical skill, our regularity detection task is geared toward our ability to perceptually track as well as produce a roughly regular rhythm, like that in speech. We therefore propose that the regularity detection threshold reflects the capacity of the brain to facilitate the structured intake and output of speech by providing a “temporal scaffolding” for both the perceptual and the motor domain (Ivry, 1996; Tierney and Kraus, 2016). One recent piece of evidence consistent with such a shared “temporal scaffolding” mechanism is the finding of rhythm perception deficits in children who stutter (Wieland et al., 2015). In previous work we have shown a much stronger correlation with reading for the regularity detection task than for the more musically relevant task of strongly metrical sequence processing accuracy (Grube et al., 2013).

### Relationship with musical expertise

The present main finding confirms the relevance of auditory regularity processing in reading skill. It is proposed that this occurs through a “temporal scaffolding” mechanism to structure input and output in time. With the observed correlation between rapid reading and regularity scores comes an effect of musical expertise that accounts for part of the correlation between reading and regularity detection. Rhythm cognition is expected to correlate with musical expertise, as musical training is thought to improve perception and production of rhythm [although recent behavioral work on the effect of formal training on different musical instruments showed no difference in superior rhythm perception and production performance between pianists, drummers, violinists and singers compared non-musicians (Matthews et al., 2016)]. The direct correlation between musical expertise and RAN times was moderate, and of borderline statistical significance. However, whether this effect is truly independent of the correlation between rhythm cognition and reading, or can be seen as an important contributing factor, remains open for further investigation. Furthermore, the causality of correlation cannot be gleaned from a cross-sectional study: the relationship with musical expertise could in part be due to a predisposition to take up music lessons if one has a good feeling for rhythm. Either way, the moderate effects found for our fairly crude measure of musical expertise in a relatively small sample, suggests that the true effect may well be stronger than observed.

The effect of musical expertise lends support to the shared resources model (e.g., Patel, 2003; Gordon et al., 2011) and is in line with a number of other groups' work on correlations between language and literacy skill, and more musically oriented rhythm tasks (e.g., Overy, 2003; Huss et al., 2011; Strait et al., 2011, 2013). This effect is somewhat consistent with the findings of our previous study (Grube et al., 2013) in which we used a number of word, non-word and poetry reading measures. We demonstrated there a strong effect for regularity detection, followed by a moderate one for strongly metrical rhythm processing, but none for that of gradual tempo contour of similar complexity to the regularity detection task. Taken together, the existing findings support the interpretation that the ability to analyze temporal structures with a quasi-regular beat is particularly relevant to speech and language skills. Musical training in turn may improve rhythm cognition abilities in a top-down fashion, and thereby strengthen the behavioral link between reading and regularity detection, as seen in the present work and supported by a recent report of neural correlates of enhanced speech rhythm sensitivity and musical aptitude (Magne et al., 2016). Overall, the evidence that music and speech, two sophisticated “high-end applications” of human auditory processing, have common underlying brain mechanisms seems strong, and is further corroborated by shared patterns of learning and brain plasticity in the two domains (see review by Zatorre, 2013). The extent to which these processes, and the mechanisms they employ to analyze rhythm, are shared or specialized will be the subject of further work (c.f. Strait et al., 2011, 2013).

### Cross-lingual universality

The observed correlation holds across different languages, as tested here in a mixed cohort of twenty-six adult native speakers of eleven languages. We find a strong and significant relationship between the participants' abilities to detect a roughly regular beat and to rapidly read out loud a list of 50 digits in their mother tongue. The participants were all fairly highly educated, and all rated themselves as advanced speakers of at least one additional language (Table 1). One might hypothesize an effect of linguistic background on RAN performance or regularity detection, or a correlation between the two. We therefore conducted additional analyses to test for such effects (using the number of second languages spoken proficiently) but did not find even a trend in the hypothesized direction. It cannot be known whether the absence of an effect might be due to the crudeness of the measure or the absence of an effect, i.e., supporting the universal nature of the link between RAN and regularity detection. This will be subject to larger, systematic studies looking at this correlation in a language-background specific way. Whilst the scope of this study did not allow for a comparative mother-tongue specific analysis, there were no clear deviations as a function of language. Consistent with this, a 10-month longitudinal study (from the start of formal literacy instruction) by Caravolas et al. (2012) supported the RAN measure, alongside phoneme awareness and letter-sound knowledge, to tap cognitive processes that are important for learning to read in languages of all alphabetic orthographies. The authors tested this in English, Spanish, Slovak, and Czech: four languages that vary in rhythmic properties to a comparable extent with the present range of Indo-European languages with phonetic (primarily Roman) scripts. Whilst there have been demonstrations of language-specific differences in speech rhythm (Dauer, 1983; Grabe and Low, 2002; Das et al., 2008) and there is an ongoing search for metrics to best capture them (Patel et al., 2006; Nolan and Asu, 2009; Turk and Shattuck-Hufnagel, 2013; Dellwo et al., 2015), we would argue that the presence of a roughly regular beat of some kind is inherent to them all. Consistent with our cross-lingual finding for rhythmic regularity processing, Goswami and coworkers have demonstrated a universal role for aspects of sound rise-time as a fundamental, language-general feature. Specifically, Muneaux et al. (2004) reported deficits in cyclic amplitude modulation in French-speaking dyslexic compared to typically developing children, and Goswami et al. (2011) showed a corresponding consistent weakness in the sensitivity to the rate of onset of the amplitude envelope (rise time) in English, Spanish, and Chinese, three languages with distinct rhythmic properties. Taken together, cross-lingual work undertaken here and elsewhere will be important in informing the design of training strategies for language development, regardless of language-specific differences in phonemes (Näätänen et al., 1997), speech rhythm and melody.

### Language impairment

The present data demonstrate a clear and strong cross-lingual correlation between the processing of rhythmic regularity of generic, pre-phonemic tone sequences, and normal adult reading skill. Whether this correlation will hold in individuals with language impairments remains to be explored. Based on other groups' reports on auditory and specifically rhythm deficits (discussed above), and our finding of the same correlation with shorter, simpler rhythm processing tasks in 11-year olds with typical development (Grube et al., 2012) and dyslexic traits (Grube et al., 2014), we would expect the present correlation to be found in listeners with language impairments. Given that correlations in our studies are stronger for more generic (i.e., less musically oriented) rhythms, we hypothesize that a rhythm cognition training programme based on such “roughly” regular rhythms could be at least as efficient as musical intervention (e.g., Overy, 2003; Moreno et al., 2009; Schön and Tillmann, 2015; Habib et al., 2016), as it may tap more directly into the shared underlying mechanism.

## Conclusion

The present results support a universal, cross-lingual role for rhythmic regularity processing in adult language (specifically, rapid automated reading) skill. The strong and robust correlation with the ability to detect a “roughly” regular beat similar to the “pseudo-regular” rhythm of speech on the one hand, and the absence of such a correlation for the detection of small deviations from a perfectly regular beat on the other, suggest a differential relevance to higher order speech and language skills, reflecting an evolutionary effect manifest in individual development.

## Author contributions

AB carried out the piloting of tasks and the behavioral testing, and contributed to the writing of the manuscript. TC contributed to the design and programming of the tasks, and the writing of the manuscript. MG designed the work, programmed the tasks, supervised the data acquisition, analyzed and interpreted the data, and wrote the manuscript.

## Funding

The research leading to these results has received funding from the People Programme (Marie Curie Actions) of the European Union's Seventh Framework Programme (FP7/2007-2013) under REA grant agreement no. 600209 (IPODI fellowship awarded to MG). Author TC was supported by the UK National Institute for Health Research (NIHR), the Association of British Neurologists, and the Patrick Berthoud Charitable Trust.

### Conflict of interest statement

The authors declare that the research was conducted in the absence of any commercial or financial relationships that could be construed as a potential conflict of interest.
