# Supplementary material for: The Beat to Read: A Cross-Lingual Link between Rhythmic Regularity Perception and Reading Skill
Source: Front Hum Neurosci. 2016 Aug 31;10:425. doi: 10.3389/fnhum.2016.00425 (PMC5006315; doi:10.3389/fnhum.2016.00425)
Supplement: Supplementary file 1 [file DataSheet1.DOCX]

**Supplementary Material**

**The Beat to Read:
A Cross-lingual Link between Rhythmic Regularity Perception and Reading Skill.**

Annike Bekius^a,b^, Thomas E Cope^c,d^, Manon Grube^a,c*^

**Material and Methods.**

***Example Stimuli*.**  We provide here example stimuli at different levels of irregularity, ranging from 0% jitter (perfectly regular) to 30% jitter (highly irregular). Note that, for clarity in listening comparison, all example stimuli consists of 10 tones each, and have an underlying tempo of 400 ms. Experimental stimuli varied between 9, 10 and 11 tones per sequence, and between three different tempos of 340, 400 and 460 ms, respectively. This was to prevent temporal expectation or subjective rhythmic “patterning” effects in terms of the number of tones, and habituation effects with respect to the underlying tempo.

Please note that individuals thresholds vary greatly, both for *irregularity detection that is in comparison to 0% jitter* (at the *regular* end, on average 4.5% ±2.6%, i.e. just below Audio 2) and *regularity detection that is in comparison to 30% jitter* (at the *irregular* end, on average 18.3% ± 4.5% i.e. between Audio 3 and 4).

**Audio 1:** 0% jitter, perfectly regular and perceptible as clearly regular by all participants.

**Audio 2:** 7.5% jitter, slightly irregular and perceptible as that by most people but not everyone.

**Audio 3:** 15% jitter (mid-way within the range used in this experiment), irregular and clearly perceptible as such by all participants; some individuals start finding it hard to hear the different to 30% (maximum jitter) for this already.

**Audio 4:** 22.5% jitter, very irregular and perceptible as such by all participants; most individuals find this difficult to distinguish from 30% (maximum).

**Audio 5:** 30% jitter, highly irregular (maximum within the range used) and perceptible as such by all participants.
